# P-2166. Sensitivity and Specificity of Nanopore Sequencing for Detection of Methicillin-Resistant *Staphylococcus aureus and* Methicillin-Resistant *Staphylococcus epidermidis* in Positive Blood Culture Samples

**DOI:** 10.1093/ofid/ofae631.2320

**Published:** 2025-01-29

**Authors:** Onjira Mangkalamanee, Kornthara Kawang, Pornsawan Cholsaktrakool, Supakorn Maita, Tongsorn Prasertmanakit, Kraiwit Pollapong, Chalurmpon Srichomthong, Vorasuk Shotelersuk, Napawan Punakabutra, Tanittha Chatsuwan, Voraphoj Nilaratanakul

**Affiliations:** Hatyai Hospital, Songkhla, Thailand, Bangkok, Krung Thep, Thailand; Faculty of Medicine, Chulalongkorn University and King Chulalongkorn Memorial Hospital, Bangkok, Krung Thep, Thailand; Program in Bioinformatics and Computational Biology, Graduate School, Chulalongkorn University, Bangkok, Krung Thep, Thailand; Faculty of Allied Health Sciences, Burapha University, Bangkok, Krung Thep, Thailand; Faculty of Allied Health Sciences, Burapha University, Bangkok, Krung Thep, Thailand; Medical Microbiology Interdisciplinary, Graduate school, Chulalongkorn University, Bangkok, Krung Thep, Thailand; Faculty of Medicine, Chulalongkorn University and King Chulalongkorn Memorial Hospital, Bangkok, Krung Thep, Thailand; Faculty of Medicine, Chulalongkorn University and King Chulalongkorn Memorial Hospital, Bangkok, Krung Thep, Thailand; King Chulalongkorn Memorial Hospital, Bangkok, Krung Thep, Thailand; Department of Microbiology, Faculty of Medicine, Chulalongkorn University, Bangkok, Krung Thep, Thailand; Division of Infectious Diseases, Department of Medicine, King Chulalongkorn Memorial Hospital, Thailand, Krung Thep, Thailand

## Abstract

**Background:**

Nanopore sequencing enables rapid DNA/RNA analysis without amplification. Implementing this for point-of-care diagnostics reduces time delays in traditional AST methods, potentially enhancing patient outcomes. We aim to evaluate the performance of Oxford Nanopore Technologies sequencing from positive blood culture for bacterial identification and antimicrobial susceptibility prediction in *S. aureus* and *S. epidermidis* by detecting the *mecA* gene.

**Figure:**
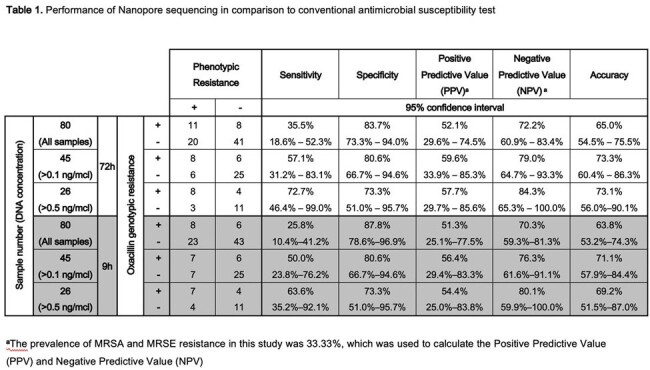

**Methods:**

We conducted a cross-sectional descriptive study on individuals with positive blood cultures identifying *S. aureus* and *S. epidermidis* over a 12-month period from April 2023 to March 2024. Bacterial DNA was extracted from the blood culture specimens, and then sequenced using the PromethION Flow Cell to identify the organisms and *mecA* genes, comparing the results to routine blood cultures and standard AST.

**Figure:**
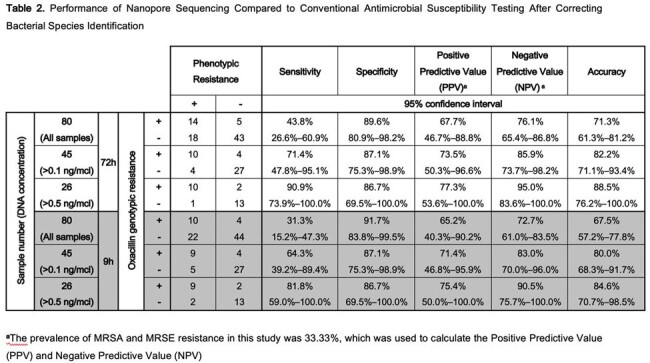

**Results:**

108 blood culture samples were initially collected. After applying the exclusion criteria, 80 samples qualified for DNA extraction and sequencing. 45 samples were identified as *S. aureus* (6 MRSA and 39 MSSA) and 35 as *S. epidermidis* (27 MRSE and 8 MSSE). Notably, 67.5% of samples contained less than 0.5 ng/μl of DNA. Sensitivity and specificity of *mecA* gene detection were 35.5% (95% CI: 18.6%–52.3%) and 83.7% (95% CI: 73.3%–94.0%), respectively, with an accuracy of 65.0% (95% CI: 54.5%–75.5%). Upon excluding samples with DNA less than 0.5 ng/μl accuracy improved to 73.1% (95% CI: 56.0%–90.1%). The specimen that showed a false-positive result will proceed to phenotypic confirmation using methicillin-infused mannitol-salt agar. After phenotypic confirmation, the accuracy improved to of 88.5% (95% CI: 76.2%–100.0%). In this study, we also found 5 samples in which the *mecA* gene was shown to be genotypically oxacillin-sensitive after phenotypic confirmation. The median time for detecting the *mecA* gene was 198 minutes (IQR: 37.5–458.7) after the onset of sequencing.

**Conclusion:**

Our study on nanopore sequencing for MRSA and MRSE detection from blood cultures found promising but limited sensitivity and specificity in detecting *mecA* gene. Challenges in DNA extraction from Gram-positive bacteria affected overall accuracy. Further improvements are needed for enhanced diagnostic reliability.

**Disclosures:**

All Authors: No reported disclosures

